# Identification and characterization of catalase genes involved in the response to heat stress in *Tetranychus urticae* (Acari: Tetranychidae)

**DOI:** 10.1186/s12864-025-12215-3

**Published:** 2025-11-18

**Authors:** Bin Wei, Yue Wang, Xuechun Liu, Pengliang Yu, Yang Liu, Jingwen Shao, Suqin Shang, Youssef Dewer

**Affiliations:** 1 College of Plant Protection, Biocontrol Engineering Laboratory of Crop Diseases and Pests of Gansu Province, Gansu Agricultural University, Lanzhou, 730070 China; 2 Phytotoxicity Research Department, Central Agricultural Pesticide Laboratory, Agricultural Research Center, 7 Nadi El-Seid Street, Dokki, Giza, 12618 Egypt

**Keywords:** Tetranychus urticae, Catalase, Heat stress, RNA interference

## Abstract

**Background:**

As global temperatures rise, organisms must adapt to heat stress to survive. Heat stress induces the production of reactive oxygen species (ROS), which can damage cells and increase mortality. Catalase (CAT), an antioxidant enzyme, mitigates this damage by breaking down hydrogen peroxide, a major ROS.

**Results:**

In this study, we identified and characterized two catalase genes, *TuCAT1* and *TuCAT2*, from the transcriptome of *Tetranychus urticae*, a pest species known for its resilient to environmental stress. Sequence analysis showed that both CAT proteins are highly conserved across arthropods, with TuCAT1 and TuCAT2 closely related to the CAT protein of *Panonychus citri*. Under heat stress, *TuCAT1* gene expression was significantly upregulated, while *TuCAT2* was down-regulated, indicating a complex regulatory response. RNA interference (RNAi) silencing of *TuCAT1* enhanced superoxide dismutase and peroxidase activity but decreased survival under heat stress.

**Conclusion:**

These findings deepen our understanding of catalase’s role in heat tolerance in *T. urticae* and guiding pest management strategies in the context of climate change.

**Supplementary Information:**

The online version contains supplementary material available at 10.1186/s12864-025-12215-3.

## Background

*Tetranychus urticae* Koch, commonly known as the two-spotted spider mite, is one of the most destructive pest mites worldwide, feeding on more than 1,200 plant species across 140 families, including crops, fruit trees, vegetables, ornamental plants, forest species, and medicinal herbs [1]. The rapid expansion of modern agricultural systems—particularly controlled-environment agriculture such as greenhouses—has created optimal conditions for crop growth, increasing productivity and land use efficiency [2]. However, these enclosed environments often experience elevated temperatures, frequently exceeding 40 °C, which are highly favorable for the proliferation of *T. urticae*, a species known for its strong tolerance to heat and drought [3]. As a result, mite populations can rapidly multiply, causing significant damage in controlled agricultural systems.

As ectothermic organisms, mites like *T. urticae* have limited ability to regulate their internal body temperature and are therefore highly sensitive to ambient thermal fluctuations [4, 5]. During aerobic metabolism, reactive oxygen species (ROS) are naturally generated and typically balanced by the organism’s antioxidant defense systems [6]. Under normal physiological conditions, ROS play important roles in cell signaling, immunity, and development [7, 8]. However, environmental stressors such as extreme temperatures, pesticide exposure, or heavy metals can cause excessive ROS accumulation, leading to oxidative stress, cellular damage, DNA mutations, and increased mortality [9–12].

To mitigate the harmful effects of ROS, organisms rely on a suite of antioxidant mechanisms, including both enzymatic and non-enzymatic defenses [13, 14]. Among these, superoxide dismutase (SOD) converts superoxide radicals into hydrogen peroxide (H₂O₂), which is then further broken down into water and oxygen by catalase (CAT), making CAT a key enzyme in the detoxification pathway [15].

Numerous studies have demonstrated that heat stress significantly influences CAT activity and gene expression. For instance, in *Chilo suppressalis*, exposure to temperatures above 30 °C resulted in a 6.2–9.5-fold increase in both CAT activity and gene expression [16]. Similar responses were observed in *Aphelinus asychis* and *Bemisia tabaci*, with CAT gene expression peaking at high temperatures and prolonged heat exposure, while CAT gene silencing in *B. tabaci* significantly reduced survival under heat stress [17, 18]. Other arthropods such as *Myzus persicae*, *Mononychellus mcgregori*, *Neoseiulus cucumeris*, and *Neoseiulus barkeri* also exhibit upregulated CAT activity and gene expression in response to heat stress [6, 19–22]. In *T. urticae*, CAT activity was shown to increase 2.4-fold after 2 h at 39 °C [23]. However, the specific transcriptional responses of CAT genes to short-term heat stress in this species remain poorly understood.

This study aimed to investigate the role of the CAT gene family in *T. urticae* under short-term heat stress. We cloned and characterized two catalase genes, *TuCAT1* and *TuCAT2*, and predicted their protein features using bioinformatics tools. Gene expression patterns under varying heat conditions and time points were assessed using real-time quantitative PCR (RT-qPCR). Furthermore, RNA interference (RNAi) was applied to silence the *TuCAT* genes and evaluate their functional roles under heat stress conditions. The results of this study provide new insights into the molecular mechanisms underlying thermal tolerance in *T. urticae* and may support the development of more effective pest management strategies in the context of global climate change.

## Methods

### Mite culture

The *Tetranychus urticae* population used in this study was originally collected from Xinlong Mountain, Gansu Province, China, in May 2012. The mites were subsequently reared for over 40 generations on *Phaseolus vulgaris* leaves without pesticide exposure. Rearing was conducted in a controlled environment chamber at the Department of Insect Systematics and Biodiversity, College of Plant Protection, Gansu Agricultural University, China [24]. The rearing conditions were set as: temperature of 25 ± 1 °C, 60–70% relative humidity (RH), and a photoperiod of 16/8 hours.

### Screening of catalase genes

Genetic screening of catalase genes was conducted based on transcriptome data of *Tetranychus urticae* (Accession number: PRJNA1073827). Gene function annotations from this dataset were used to identify and characterize catalase genes in *T. urticae*.

### Cloning the CDSs of catalase genes

The yield and quality of RNA, as well as the accuracy of the coding DNA sequences (CDS) of the target genes, are critical for successful gene cloning. CDS sequences of the catalase genes were confirmed using the Open Reading Frame (ORF) Finder tool available through NCBI (https://www.ncbi.nlm.nih.gov/orffinder/). Specific primers for amplifying the CDS regions were designed using Primer3 Input software (https://bioinfo.ut.ee/primer3/) (Table S1).

More than 300 adult female *T. urticae* mites were collected into 1.5 ml RNase-free centrifuge tubes, flash-frozen in liquid nitrogen, and stored at − 80 °C. Total RNA was extracted using RNAiso Plus reagent (Takara, Dalian, China), and RNA concentration and purity were assessed with a NanoPhotometer-N50 spectrophotometer (GE Healthcare, Wiesbaden, Germany). First-strand cDNA synthesis was performed using the PrimeScript™ II 1 st Strand cDNA Synthesis Kit (Takara, Dalian, China). PCR amplification was carried out using PrimeSTAR^®^ Max DNA Polymerase (Takara, Dalian, China). PCR products were visualized by 1% agarose gel electrophoresis, and target bands were excised and purified using the TaKaRa MiniBEST Agarose Gel DNA Extraction Kit Ver. 4.0 (Takara, Dalian, China).

The purified PCR products were ligated into the pLB-T vector (TIANGEN, Beijing, China) and transformed into *Escherichia coli* TOP10 competent cells (TIANGEN, Beijing, China). Plasmid DNA was extracted using the GeneJET Plasmid Miniprep Kit (Thermo Scientific, Lithuania, EU), and positive clones were sequenced by Sangon Biotech Co., Ltd. (Shanghai, China). The cloned CDS sequences were aligned with the *T. urticae* reference genome (GenBank accession: GCA_000239435.1) to verify their accuracy [25].

### Bioinformatic analysis of *T. urticae *CAT protein sequences

The ORF lengths of the cloned CAT protein sequences were determined using the ORF Finder tool available on the NCBI website (https://www.ncbi.nlm.nih.gov/orffinder/). Functional domains and conserved structural sites within the CAT sequences were analyzed using the Protein Family Database (https://www.ebi.ac.uk/interpro/search/sequence/) and the Conserved Domain Database via the NCBI CD-Search tool (https://www.ncbi.nlm.nih.gov/Structure/cdd/wrpsb.cgi).

CAT protein sequences from other arthropod species were retrieved from the NCBI database for comparative analysis. Multiple sequence alignments were performed using GENEDOC v2.7. To construct the phylogenetic tree, we first selected the most suitable model in MEGA 7 using the Find Best DNA/Protein Models function. Based on the results, we then used the Maximum Likelihood (ML) method in the same software to construct the phylogenetic tree, performing 1000 bootstrap replications to confirm its reliability. Finally, we imported the tree into the Adobe Illustrator 2022 to design and beautify it.

The physicochemical properties of the TuCAT proteins were analyzed using the ExPASy ProtParam tool (http://web.expasy.org/protparam). Functional and structural protein motifs were identified using the Prosite Scan tool (http://npsa-pbil.ibcp.fr/cgi-bin/npsa_automat.pl?page=/NPSA/npsa_proscan.html). Transmembrane regions and hydrophilicity profiles were predicted using TMHMM 2.0 (https://services.healthtech.dtu.dk/services/TMHMM-2.0/) and ExPASy Protscale (http://web.expasy.org/protscale), respectively. Subcellular localization predictions for the CAT proteins were performed using WOLF PSORT (https://wolfpsort.hgc.jp/). Secondary structures were predicted using SOPMA (https://npsa-prabi.ibcp.fr/cgi-bin/npsa_automat.pl?page=/NPSA/npsa_seccons.html), while three-dimensional models of the TuCAT proteins were generated using the SWISS-MODEL homology modeling tool (https://swissmodel.expasy.org/).

### Transcript expression of *T. urticae* catalase genes

A preliminary experiment was conducted in order to ascertain the effect of heat stress on the mortality rate of *T. urticae*. The findings demonstrated that at temperatures of 42 °C, the mites exhibited a significant decline in viability, with an increase in the duration of the heat stress resulting in a corresponding increase in mortality. It is imperative to ensure the survival of test mites during the experiment and to fully simulate field conditions. To this end, the formal experiment selected 36, 39, 42, and 45 °C as treatment temperatures and 2, 4, and 6 h as heat treatment durations.

*Phaseolus vulgaris* leaves infested with *Tetranychus urticae* were exposed to heat stress treatment at temperatures of 36 °C, 39 °C, 42 °C, and 45 °C, with each treatment temperature further divided into three heat stress treatment durations (2, 4, and 6 h). Mites maintained at 25 °C served as the control group. Following each heat stress treatment, *T. urticae* were collected in 1.5 ml sterile, enzyme-free centrifuge tubes and immediately snap-frozen in liquid nitrogen. Total RNA was extracted from each sample, with three biological replicates per treatment.

The extracted RNA was reverse transcribed into cDNA using the PrimeScript™ RT Reagent Kit with gDNA Eraser (Takara, Dalian, China). RT-qPCR primers were designed using Primer3, based on the cloned *TuCAT* gene sequence (Table S1). The α-tubulin gene (GenBank Accession No. JN881327.1) was used as an internal reference gene [26]. RT-qPCR was performed on an ABI QuantStudio 5 Real-Time PCR System with the TB Green Premix Ex Taq™ II (Tli RNaseH Plus) Kit (Takara, Dalian, China). Primer specificity and amplification efficiency were confirmed by melting curve analysis. Each treatment was performed in triplicate. The relative expression levels of the *TuCAT* genes were calculated using the 2^−ΔΔCt^ method.

### RNA interference

Based on the RT-qPCR results, the *TuCAT* genes that were up-regulated under short-term heat stress were selected for RNA interference (RNAi) assays. Specific primers, including the T7 RNA polymerase promoter sequence, were designed to amplify the target *TuCAT* gene. The synthesized double-stranded RNA (dsRNA) was purified and then transcribed using the TranscriptionAid T7 High Yield Transcription Kit (Thermo Scientific, Lithuania, EU).

Following the protocol described by Shen et al. [27], the dsRNA (at a concentration of 1000 ng/µl) was introduced into *Phaseolus vulgaris* leaf discs. Three hundred female adult *T. urticae* mites, which had been starved at room temperature for 24 h, were then placed onto the treated leaf discs. The leaf discs were maintained at 25 ± 1 °C with 60–70% relative humidity and a 16 L/8D photoperiod for four days, with the dsRNA-treated leaves replaced every two days. To control for experimental biases, dsRNA targeting the green fluorescent protein (*GFP*) gene was used as a negative control, and RNase-free ddH_2_O served as a blank control. Each treatment was performed in triplicate, with additional replicates as needed. Gene silencing efficiency was verified through RT-qPCR.

Upon successful silencing of the target *TuCAT* genes, the total antioxidant capacity and activities of various antioxidant enzymes were measured using commercial enzyme activity kits. Viable *T. urticae* mites that had undergone gene silencing were then exposed to heat stress at 25 °C and 42 °C for 6 h. dsGFP interference was used as a negative control, and RNase-free ddH_2_O served as a blank control. Mite mortality rates were recorded at hourly intervals.

### Statistical analysis

The present study utilised R software (version 4.5.1, R Foundation for Statistical Computing) to conduct a statistical analysis of the expression levels of the *CAT* genes in *T. urticae* under various temperature and time treatments. The data violated the key assumptions of parametric analysis (normality of residuals and homogeneity of variances), as confirmed by Shapiro-Wilk and Levene’s tests. Consequently, a non-parametric Aligned Rank Transform (ART) procedure was employed to analyze the factorial design [28].

The transformed data were then subjected to a mixed-effects ANOVA using the ARTool package to evaluate the main effects of Factor temperatures and Factor times, as well as their interaction effect. For any significant effects identified by the ART ANOVA (α = 0.05), post-hoc analyses were conducted using pairwise Dunn’s tests with Bonferroni correction to control the family-wise error rate.

The results after RNA interference were also analyzed using paired *t*-tests with SPSS 27.0 software. Statistical significance was determined with a *p*-value threshold of < 0.05. The significance levels were indicated as follows: *p* < 0.05 (*), *p* < 0.01 (**), *p* < 0.001 (***), and ns for *p* ≥ 0.05 (not significant).

## Results

### Verification of catalase gene cloning accuracy in *T. urticae*

Using transcriptome data from *T. urticae*, we successfully cloned two catalase genes, designated *TuCAT1* and *TuCAT2*, and deposited their sequences in the NCBI database (GenBank numbers. PQ566588 and PQ566589). To verify the accuracy of these cloning results, we compared the cloned sequences with the reference genome of *T. urticae* available in the NCBI database. The comparison revealed a 99.81% sequence homology for TuCAT1 nucleotide and 100% homology for TuCAT2 nucleotide (Table 1).

**Table 1:** Homology comparison of CAT nucleotides cloned from *T. urticae*

Name	Description	Query Cover	E-Value	Identity
TuCAT1	XP_015791012.1 Catalase [*Tetranychus urticae*]	100%	0.0	99.81%
TuCAT2	XP_015789519.1 Catalase isoform X2 [*Tetranychus urticae*]	100%	0.0	100.00%

### Bioinformatics characterization of *TuCAT1* and* TuCAT2*

The ORF lengths of TuCAT1 and TuCAT2 were determined to be 1572 bp and 1527 bp, respectively, encoding proteins of 523 and 508 amino acids. Bioinformatics analysis revealed several key characteristics of these proteins: TuCAT1 has a molecular weight of 59,045.45 Da, while TuCAT2 has a molecular weight of 57,748.19 Da. The theoretical isoelectric points of TuCAT1 and TuCAT2 are 5.96 and 6.79, respectively. Additionally, their aliphatic indices are 74.42 and 70.63. Both proteins are hydrophilic and stable, with no predicted transmembrane helical regions (Table 2, Figures S1-S4).

**Table 2:** Detailed biological information of TuCAT1 and TuCAT2

Gene	ORF	aa	Formula	Molecular Weight	Theoretical pI	Aliphatic index	Instability coefficient
*TuCAT1*	1572	523	C_2654_H_4029_N_713_ O_788_S_16_	59045.45	5.96	74.42	54.34
*TuCAT2*	1527	508	C_2589_ H_3949_ N_715_ O_757_ S_17_	57748.19	6.79	70.63	40.13

Gene	Instability coefficient	Protein Stability Prediction	Hydrophilicity Value	Protein Hydrophilicity Prediction	Protein Subcellular Localization Prediction
*TuCAT1*	35.11	Stable Protein	−0.347	Hydrophilic Protein	Extracellular
*TuCAT2*	30.16	Stable Protein	−0.551	Hydrophilic Protein	Cytoplasm

**Fig. 1:**
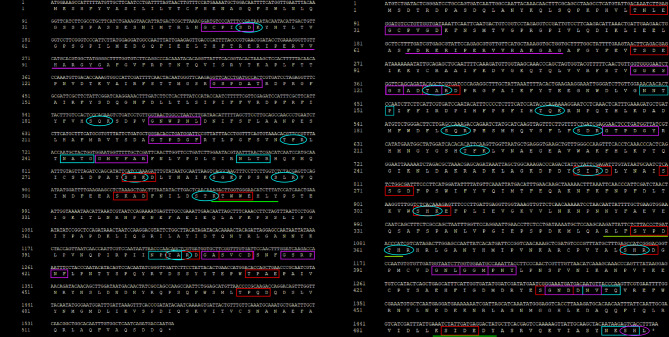
Distribution of structural sites in the catalase proteins (**A**: TuCAT1; **B**: TuCAT2) of *T. urticae*. The red-boxed section indicates N-glycosylation site, the blue-boxed section represents the casein kinase II phosphorylation site, the green-boxed section designates the N-myristoylation site, the red-rounded section highlights the protein kinase C phosphorylation site, the green-rounded section illustrates the microbodies C-terminal targeting signal, the pink-underlined section denotes the tyrosine kinase phosphorylation site, the green-underlined section represents the catalase proximal active site, and the purple-underlined section indicates the catalase proximal heme-ligand

Protein structure analysis identified several functional sites in both TuCAT1 and TuCAT2. TuCAT1 contains three N-glycosylation sites, eight protein kinase C phosphorylation sites, seven casein kinase II phosphorylation sites, one tyrosine kinase phosphorylation site, seven N-myristoylation sites, and one catalase proximal active site. TuCAT2 shares the same N-glycosylation and myristoylation sites but also includes nine protein kinase C phosphorylation sites, ten casein kinase II phosphorylation sites, one tyrosine kinase phosphorylation site, one microbodies C-terminal targeting signal, one catalase proximal heme-ligand, and one catalase proximal active site (Fig. 1).

**Fig. 2:**
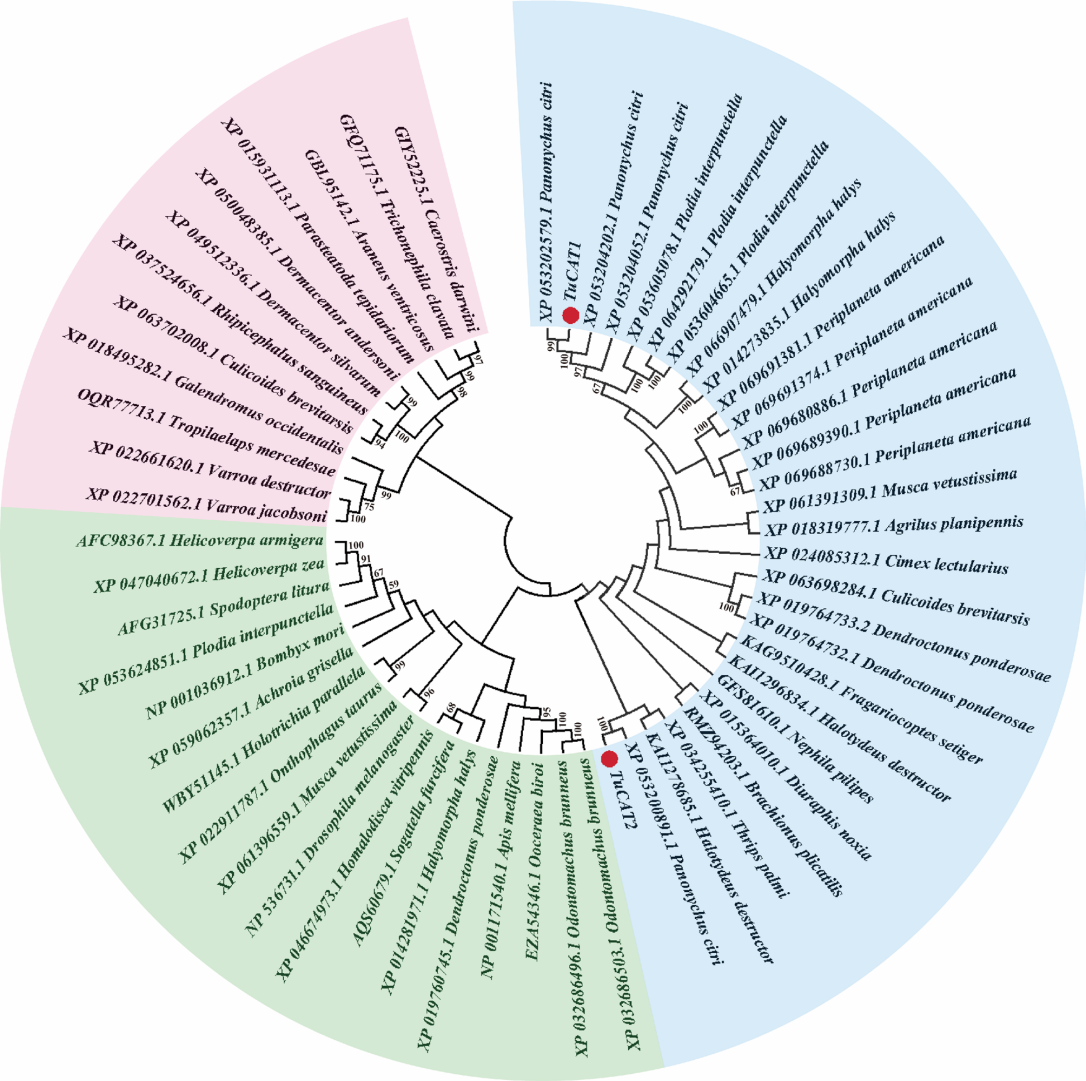
Phylogenetic relationships of the newly cloned catalase proteins in *T. urticae* and others species. *TuCAT1* and *TuCAT2* are marked with red dots. Different background colors represent different branches of the phylogenetic tree, and the numbers on the branches represent the homology ratio

**Fig. 3:**
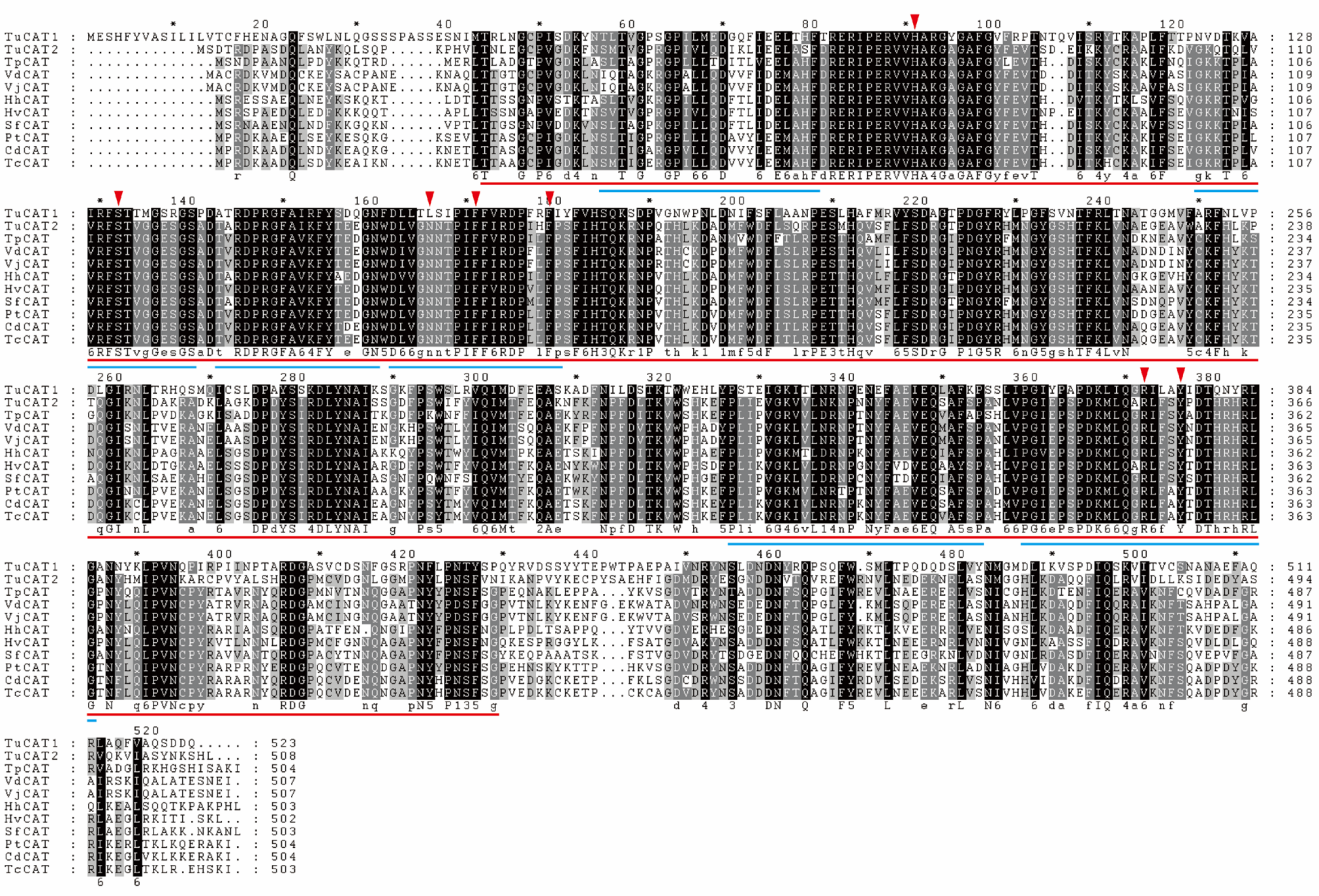
Multiple sequence alignment of the catalase proteins from *T. urticae* (TuCAT1 and TuCAT2) with other species including *Thrips palmi* (Tp), *Varroa destructor* (Vd), *Varroa jacobsoni* (Vj), *Halyomorpha halys* (Hh), *Homalodisca vitripennis* (Hv), *Sogatella furcifera* (Sf), *Parasteatoda tepidariorum* (Pt), *Caerostris darwini* (Cd), *Trichonephila clavata* (Tc). Catalase binding domains are marked with red underlines, the catalase signatures sequences are indicated with blue underlines, and the red arrow highlights the heme-binding pocket

To investigate the evolutionary relationships of TuCAT1 and TuCAT2, a phylogenetic tree was constructed, and multiple sequence alignments were performed. The analysis revealed that TuCAT1 and TuCAT2 are closely related to the CAT of *Panonychus citri* (Fig. 2). Both TuCAT sequences contain one catalase binding domain, six catalase signature sequences, and seven heme-binding pockets (Fig. 3).

**Fig. 4:**
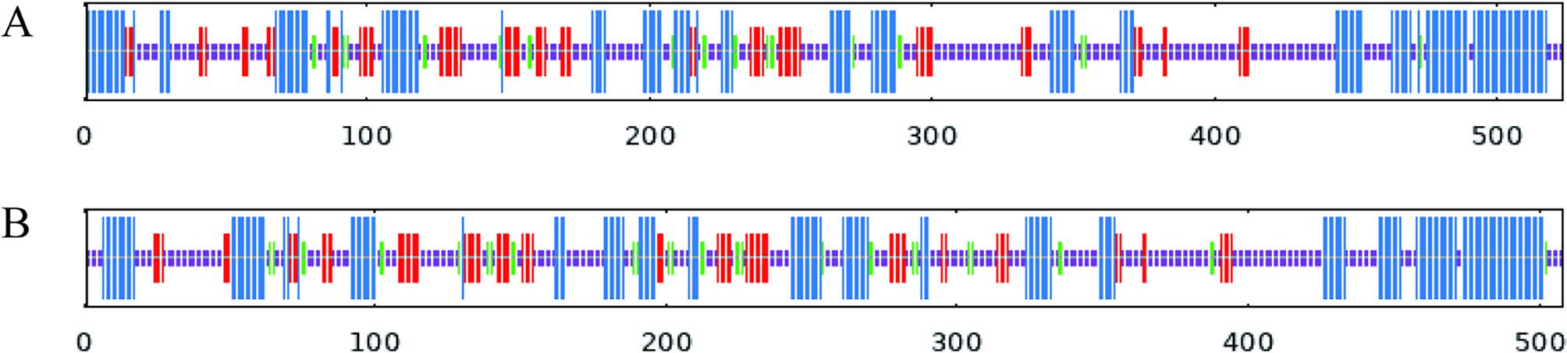
Prediction of the secondary structure of TuCAT proteins (**A**: TuCAT1; **B**: TuCAT2) in *T. urticae*. The blue segments represent alpha helices, the red segments correspond to extended strands, the green segments indicate beta turns, and the purple segments represent random coils

**Fig. 5:**
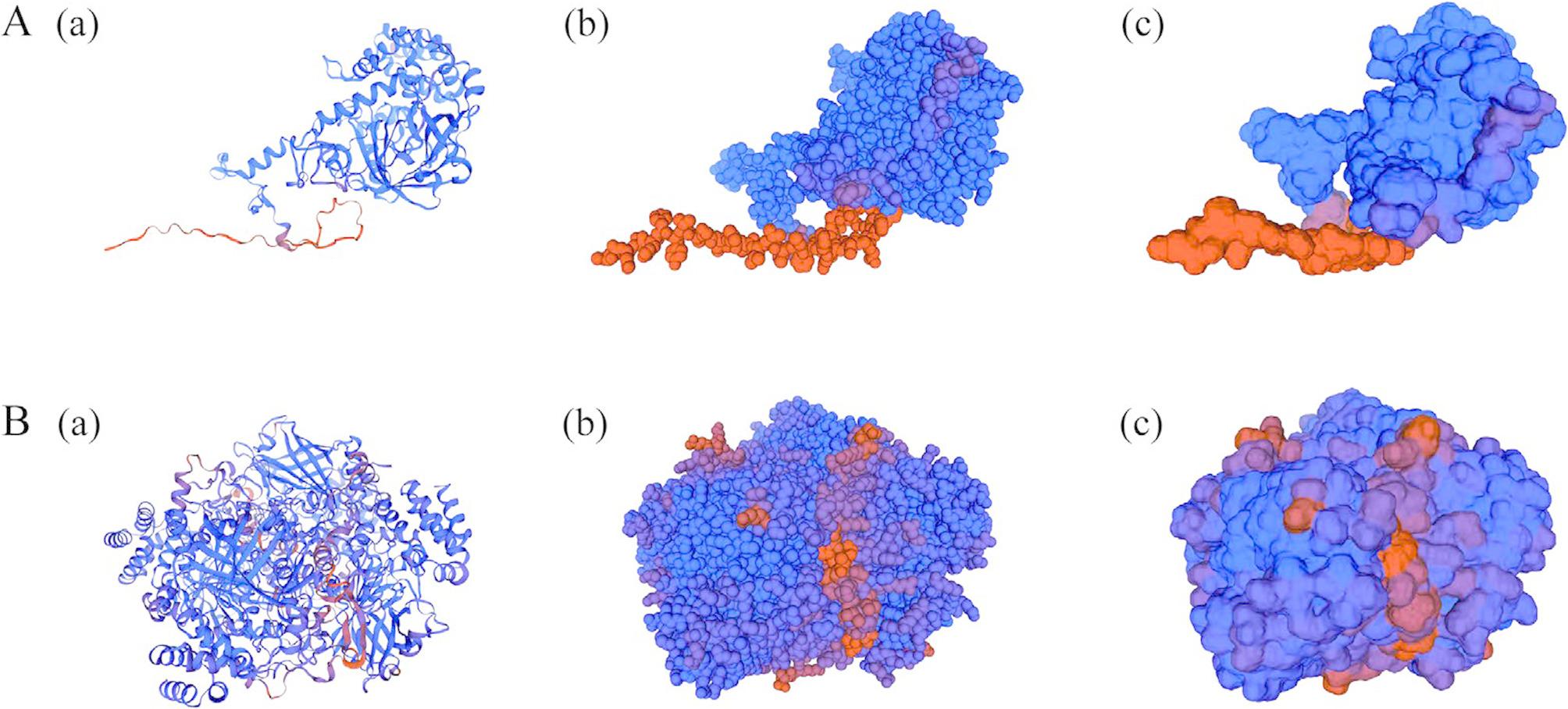
Prediction of the tertiary structure of TuCAT proteins (**A**: TuCAT1; **B**: TuCAT2) in *T. urticae*. (a) Cartoon visualization, (b) Spacefill visualization, and (c) Surface visualization

Secondary and tertiary structure predictions indicated that both TuCAT proteins are predominantly composed of random coils, accounting for 50.10% of TuCAT1 and 49.21% of TuCAT2. Alpha helices were the second most prevalent structure, comprising 30.98% of TuCAT1 and 30.12% of TuCAT2. Beta turns accounted for the smallest proportion, representing 4.40% in TuCAT1 and 6.30% in TuCAT2 (Figs. 4 and 5).

### Transcriptional expression of *TuCAT* genes under heat stress conditions

**Fig. 6:**
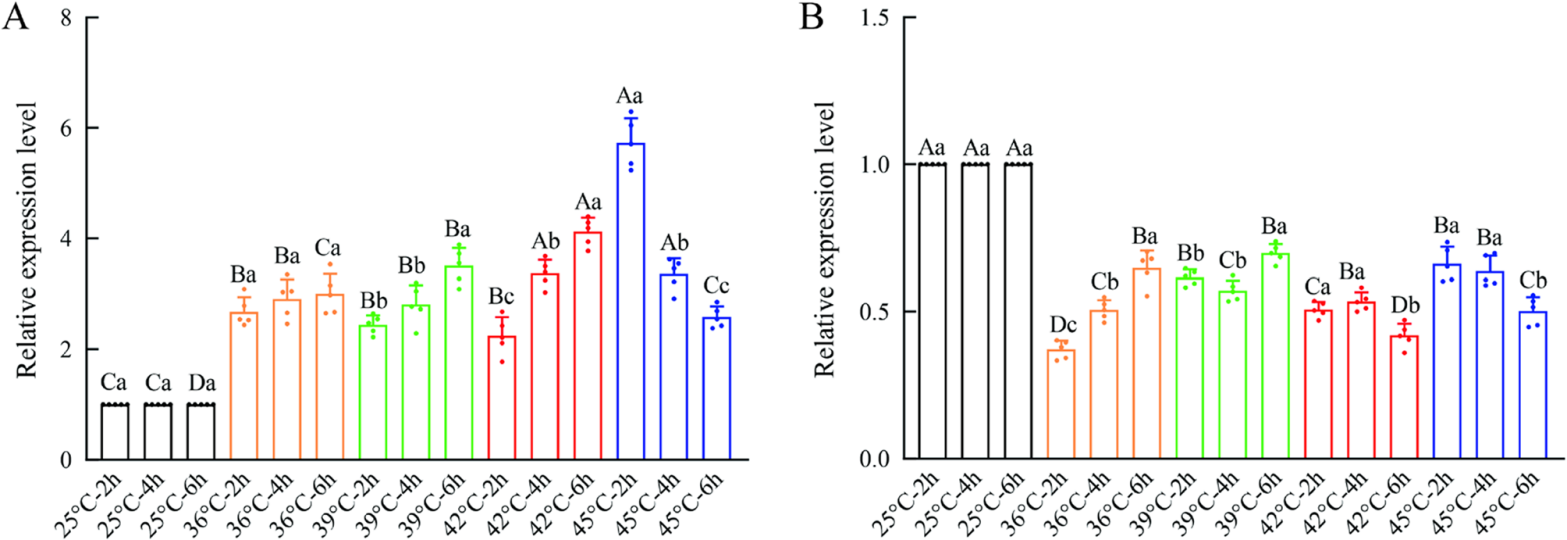
Relative expression levels of *TuCAT* genes under different heat stress temperatures and durations. **A** *TuCAT1* expression; (**B**) *TuCAT2* expression. Data are presented as mean ± SE (*n* = 5). The application of different capital letters serves to indicate significant differences in the gene relative expression level at the same heat stress times among different temperatures. Conversely, the use of lowercase letters indicates significant differences at the same heat stress temperature among different times at the 0.05 level by ART procedure

The transcriptional expression levels of *TuCAT1* and *TuCAT2* in *T. urticae* were analyzed under various short-term heat stress conditions using RT-qPCR (Table S2 and S3, Fig. 6). The results revealed *TuCAT1* expression was up-regulated under all high-temperature conditions and durations, while *TuCAT2* expression was consistently down-regulated.

The *TuCAT1* gene expression of *T. urticae* was found to be significantly affected by temperature (F = 67.05, *p* < 0.001), while the effect of time was found to be non-significant (F = 2.06, *p* > 0.01). Furthermore, the interactions among these treatment factors were found to be significant (F = 34.81, *p* < 0.01). Under heat stress at 36 °C, 39 °C, and 42 °C, *TuCAT1* expression gradually increased with longer stress durations. However, at 45 °C, expression initially increased but then decreased with prolonged exposure. After 2 h of stress, *TuCAT1* expression decreased as the temperature rose, reaching its lowest point at 42 °C. Subsequently, expression increased, peaking at 45 °C, which showed the highest expression level across all stress conditions. Under 4-hour stress, *TuCAT1* expression remained stable at 36 °C and 39 °C, with a slight increase observed at 42 °C. For the 6-hour stress duration, the expression pattern of *TuCAT1* was opposite to that observed under the 2-hour stress. Expression increased from 36 °C to 42 °C, peaking at 42 °C, and then decreased as the temperature rose further.

In comparison with the control group, the *TuCAT2* gene expression was significantly affected at all temperatures (F = 101.31, *p* < 0.01), and, conversely, was unsignificantly affected at all times (F = 2.85, *p* > 0.01). In addition, a significant interaction among these treatment factors was identified (F = 36.22, *p* < 0.01). *TuCAT2* gene expression was down-regulated under all heat stress conditions, though it reached its highest level after 6 h of exposure at 39 °C. Despite this peak, the expression remained lower than that observed in the control group at 25 °C. Under heat stress at 36 °C and 45 °C, *TuCAT2* expression exhibited temperature- and time-dependent patterns. At 36 °C, gene expression increased with longer exposure durations, while at 45 °C, expression decreased as the exposure time increased. A similar trend was observed at 39 °C and 42 °C, where *TuCAT2* expression initially decreased at 39 °C before increasing, whereas at 42 °C, expression increased initially and then declined.

When the stress duration was held constant, *TuCAT2* expression exhibited a similar trend with increasing temperatures: expression rose from 36 °C to 39 °C, decreased from 39 °C to 42 °C, and then increased again at higher temperatures. These results indicate that *TuCAT1* and *TuCAT2* exhibit distinct responses to heat stress. *TuCAT1* generally shows a positive correlation with heat exposure, while *TuCAT2* displays a more complex, temperature-dependent response.

### Physiological response of *T. urticae* to heat stress after *TuCAT1* interference

**Fig. 7:**
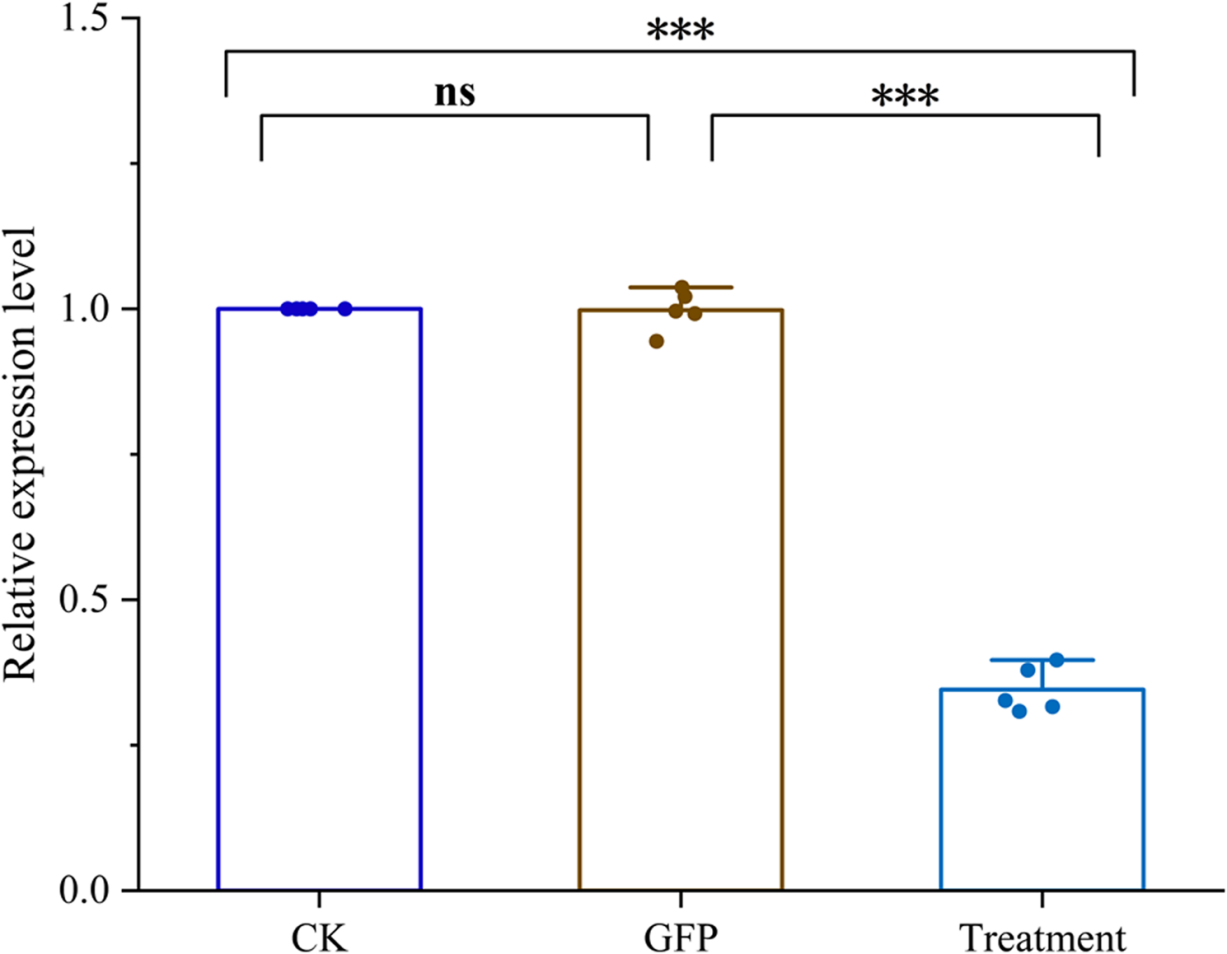
dsRNA-mediated suppression of *TuCAT1* transcript expression. The final concentration of dsRNA was 1000 ng/µL. Data are presented as mean ± SE (*n* = 5). Asterisks on the brackets indicate significant differences in expression levels among different treatments. (“*”: *p* < 0.05; “**”: *p* < 0.01; “***”: *p* < 0.001; “ns”: *p* ≥ 0.05)

The results from the last section suggest that the *TuCAT1* gene plays a critical role in the ability of *T. urticae* to manage short-term heat stress. To explore this further, we synthesized double-stranded RNA (dsRNA) targeting a partial sequence of *TuCAT1* and introduced it into *T. urticae* to achieve gene knockdown. The knockdown was successful, with a silencing efficiency of 65.46% compared to the control group (Fig. 7).

**Fig. 8:**
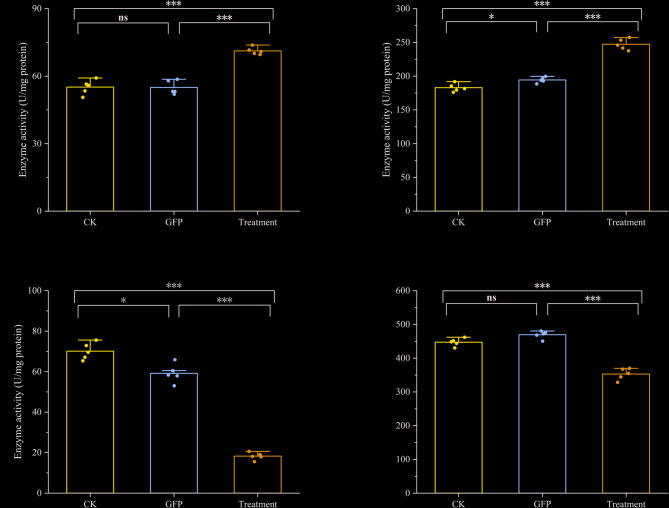
Effects of *TuCAT1* knockdown on different enzyme activities and total antioxidant capacity. **A** Superoxide dismutase activity; (**B**) Peroxidase activity; (**C**) Catalase activity; (**D**) Total antioxidant capacity. Data are presented as mean ± SE (*n* = 5). Asterisks on the brackets indicate significant differences in expression levels among different treatments. (“*”: *p* < 0.05; “**”: *p* < 0.01; “***”: *p* < 0.001; “ns”: *p* ≥ 0.05)

We then assessed the impact of *TuCAT1* interference on the antioxidant capacity of *T. urticae* by measuring the activities of key antioxidant enzymes and the total antioxidant capacity. The results showed that, in the *TuCAT1* interference group, the activities of superoxide dismutase (SOD) and peroxidase (POD) were elevated compared to both the blank control and dsGFP control group. However, both catalase (CAT) activity and total antioxidant capacity (T-AOC) were significantly reduced (Fig. 8).

**Fig. 9:**
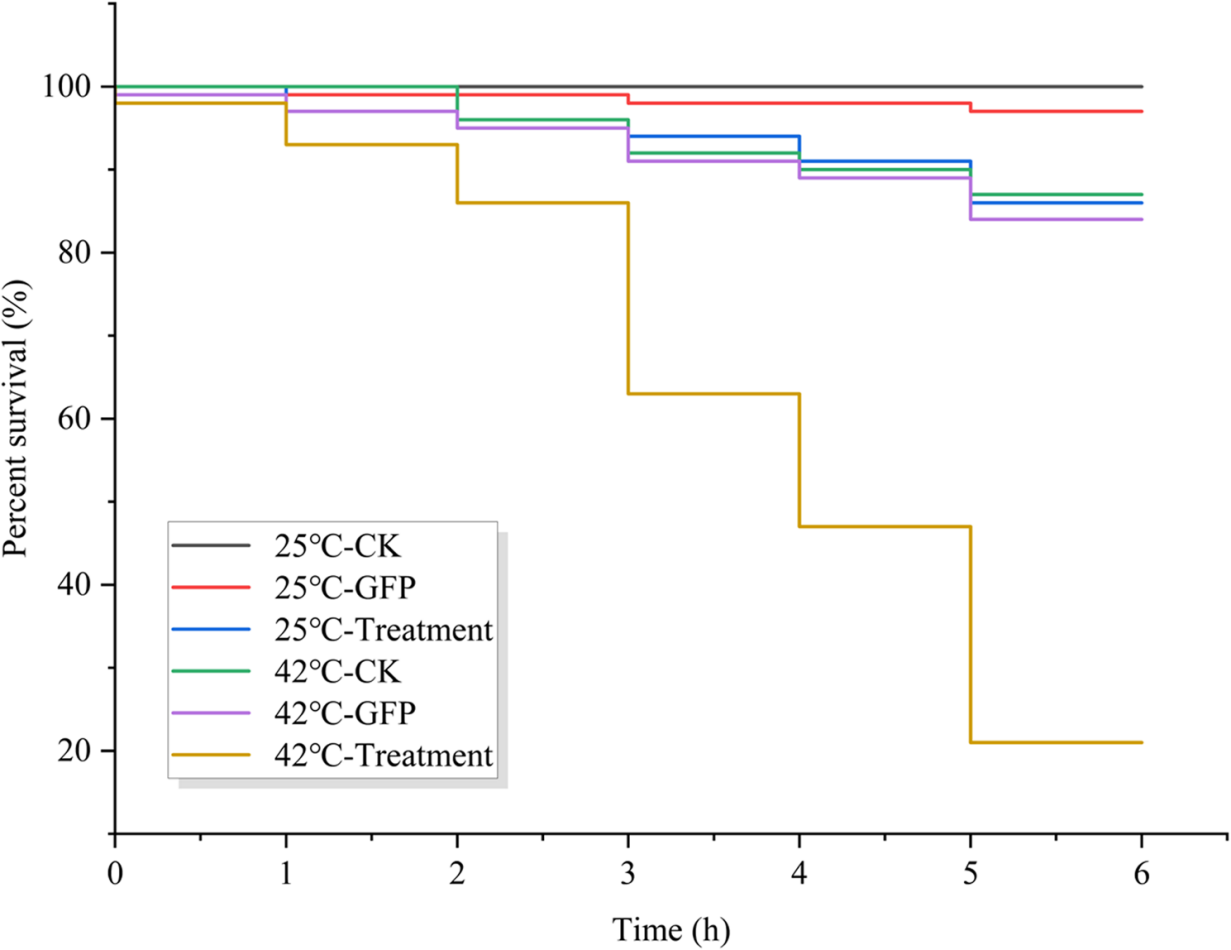
Survival of *T. urticae* under different temperatures stress after silencing of *TuCAT1* gene

Next, the survival rates of treated *T. urticae* were analysed statistically, with the ambient temperature (25 ℃) and elevated temperature (42 ℃) being used as the temperature parameters (Fig. 9). At a temperature of 25 ℃, the survival rate of mites in the blank control group remained at 100%, while those in the dsGFP control and interference groups gradually decreased over time. Following a six-hour exposure period, survival rates were recorded as 97% and 86%, respectively. At a temperature of 42 ℃, a decline in survival rates was observed across all groups over time. The blank control group and the dsGFP control group both reached 87% and 84%, respectively, while the interference group decreased to 21% after 6 h of exposure.

The survival rates of test mites were compared in the blank control group, the dsGFP control group, and the interference group at both ambient and elevated temperatures. The results demonstrated that the survival rate of test mites in the control groups was marginally lower at elevated temperatures that at ambient temperatures. A 13% discrepancy was observed between the blank control and the dsGFP control groups after six hours of exposure. By contrast, the survival rate of test mites in the interference group was 65% lower at elevated temperatures than at normal temperatures. Furthermore, it was observed that under conditions of elevated temperature, the hourly survival rate of the interference group was consistently lower than that of both control groups, and the survival rate decreased more rapidly with longer exposure times.

## Discussion

For poikilothermic animals, the ability to adapt to temperature fluctuations is crucial for survival [29]. In the face of global warming and advances in agricultural practices, *Tetranychus urticae* has shown a significant increase in reproductive rates and harmful potential [30]. To cope with the damaging effects of heat stress, *T. urticae* has evolved various behavioral and physiological strategies [21]. One such strategy involves catalase (CAT), an antioxidant enzyme that plays a critical role in neutralizing hydrogen peroxide (H₂O₂), a byproduct of elevated temperatures, thereby maintaining cellular redox balance [16]. In conjunction with superoxide dismutase (SOD), CAT helps stabilize cellular redox states. Despite its key role in the antioxidant defense system, the exact regulations by which the *CAT* gene functions under short-term heat stress in *T. urticae* remain unclear. In this study, we cloned and identified two *CAT* genes in *T. urticae*, performed bioinformatics analysis of their amino acid sequences, and explored the role of *TuCAT1* in the mite’s heat stress response.

The cDNA sequences of the *TuCAT* genes were cloned using PCR technology. The calculated molecular weights of TuCAT1 (59.045 kDa) and TuCAT2 (57.748 kDa) are consistent with those of other insect CAT proteins [31, 32]. Furthermore, the prediction results indicate the presence of N-glycosylation sites in both TuCAT1 and TuCAT2. This observation lends support to the hypothesis that these two peroxidases may function as glycoproteins, a property that is analogous to the analogous proteins in other species [33, 34]. The cloned amino acid sequences of both proteins exhibited high homology with the CAT proteins of other species, particularly *Panonychus citri*, with *TuCAT1* and *TuCAT2* showing 99% and 100% homology with the corresponding CAT genes, respectively. The disparities in *TuCAT1* and *TuCAT2* evolutionary relationships may be attributable to variations in their molecular structures [35]. The catalytic mechanism of CAT involves two key steps: the reduction of hydrogen peroxide to water by heme Fe³⁺, followed by the oxidation of another molecule of hydrogen peroxide to molecular oxygen, releasing water in the process [36, 37]. The cloning results from this study provide a solid foundation for further research into the characteristics and biological functions of CAT proteins in arachnids.

To explore the expression patterns of *TuCAT* genes under heat stress, we employed RT-qPCR to measure the expression levels of these genes at various high temperatures and stress durations. The results indicated that *TuCAT1* plays a more prominent role than *TuCAT2* in *T. urticae*’s adaptation to high temperatures. *TuCAT1* expression was significantly increased under all heat stress conditions, which is consistent with findings in *Chilo suppressalis* larvae under heat stress [16]. The fact that *TuCAT1* was the only *CAT* gene to exhibit increased expression under heat stress suggests that the two genes may have different expression patterns in various parts of the organism. We hypothesize that *TuCAT1* is more broadly expressed throughout the body, while *TuCAT2* may have a more localized expression, as observed in other species such as *Acipenser brevirostrum* [38] and *Scophthalmus maximus* [39]. However, further experiments are needed to confirm this hypothesis in *T. urticae*.

Given the challenges posed by dsRNA injections, which can cause mechanical damage and high mortality in insects [40], we opted for a feeding method to induce RNA interference (RNAi). In this study, *T. urticae* fed with *dsTuCAT1* for four days achieved a silencing efficiency of 65.46%, similar to the 64% silencing efficiency achieved by *Sitobion avenae* after six days of feeding on dsCAT [40]. This suggests that gene silencing efficiency of RNAi can vary significantly across insect species, even when targeting the same genes. The amount of dsRNA consumed plays a crucial role in the silencing efficiency, with greater intake leading to more pronounced effects, such as those on *Spodoptera exigua* [41], which suggest that dsRNA needs a certain period to reach effective reaction sites.

The interference of *TuCAT1* expression resulted in significantly increased mortality rates for *T. urticae* under heat stress, reducing its heat tolerance. Similar findings were reported in studies on *Lutzomyia longipalpis* [42] and *Anopheles gambiae* [43], highlighting the crucial role of *CAT* genes in organismal development. Silencing or inactivating CAT impairs the ability to process reactive oxygen species (ROS), thereby increasing susceptibility to H₂O₂-induced damage.

While substantial research has been conducted on pest management, selecting appropriate target genes for effective pest control remains a challenge, and the full potential of RNAi for pest elimination has yet to be realized [44]. This study provides compelling evidence for the critical role of catalase in *T. urticae*’s response to short-term heat stress. Future research should explore the molecular mechanisms underlying heat tolerance in *T. urticae*, offering valuable insights into how other spider mite species—and potentially other arthropods—respond to heat stress and other abiotic challenges. This work lays the foundation for advancing our understanding of the molecular basis of heat stress tolerance in arachnids.

## Conclusions

This study identified two catalase (CAT) genes, *TuCAT1* and *TuCAT2*, in *T. urticae* and investigated their roles in heat stress adaptation. *TuCAT1*, which showed significantly increased expression under heat stress, was found to be the key gene involved in the mite’s heat tolerance. RNA interference of *TuCAT1* led to increased mortality and reduced heat resistance, confirming its essential role in stress response. The identification of two *TuCAT* genes suggests that gene redundancy may enhance heat tolerance, contributing to the mite’s harmful potential. Overall, this research deepens our understanding of the molecular mechanisms behind heat stress tolerance in *T. urticae* and offers insights into future pest management strategies in the context of climate change.

## Supplementary Information

Supplementary Material 1.

Supplementary Material 2.

Supplementary Material 3.

Supplementary Material 4.

Supplementary Material 5.

Supplementary Material 6.

Supplementary Material 7.

## Data Availability

All relevant data support this research is included in this article and supplementary file. The sequences of two CATs from this study were submitted to GenBank with accession number PQ566588 and PQ566589.

## Abbreviations

*T. urticae*: *Tetranychus urticae*
ROS: Reactive oxygen species
H_2_O_2_: Hydrogen peroxide
CAT: Catalase
SOD: Superoxide dismutase
POD: Peroxidase
RT-qPCR: Real-time quantitative PCR
RNAi: RNA interference
RH: Relative humidity
CDS: Coding DNA sequences
ORF: Open reading frame
dsRNA: Double-stranded RNA
GFP: Green fluorescent protein
T-AOC: Total antioxidant capacity
