# Supplementary material for: Identification and characterization of catalase genes involved in the response to heat stress in Tetranychus urticae (Acari: Tetranychidae)
Source: BMC Genomics. 2025 Nov 18;26:1053. doi: 10.1186/s12864-025-12215-3 (PMC12625012; doi:10.1186/s12864-025-12215-3)
Supplement: Supplementary file 1 — Supplementary Material 1. [file 12864_2025_12215_MOESM1_ESM.pdf]

Table S1. Primer sequences used in this study

Gene name	Primer name	Primer sequences (5'-3')	Description
TuCAT1	*TuCAT1*-F	GGACTAATTGCCATTGTTCCCA	Primers for cloning
	TuCAT1-R	CTGTTTCAACCAACAAAGGATGT	
	q*TuCAT1*-F	CTTTTGGTGTCTTCAGGCCC	Primers for RT-qPCR
	q*TuCAT1*-R	CTCTAGGATCACGAGTGGCA	
	dsTuCAT1-F	**TAATACGACTCACTATAGGG**GTGATGGTGCTTCGGTTTGT	Primers for RNAi
	dsTuCAT1-R	**TAATACGACTCACTATAGGG**CCAAACTGTCCTGGTCTTGC	
TuCAT2	*TuCAT2*-F	TCTCTCCCTTTCAACAGCCA	Primers for cloning
	TuCAT2-R	CCAGAGTCGACTTGCCCAATTA	
	q*TuCAT2*-F	ACAATCAGCTTTCTCGCCAG	Primers for RT-qPCR
	q*TuCAT2*-R	ACATCGAGCTTTGTTGACCG	
α-tubulin	q*α-tubulin*-F	TCTTGTCCCCTACCCTCGTA	Primers for RT-qPCR
	q*α-tubulin*-R	TTTCCATGTCGAGGGTCACA	
GFP	*dsGFP*-F	**TAATACGACTCACTATAGGG**ATGGTGAGCAAGGGCGAGG	Primers for RNAi
	dsGFP-R	**TAATACGACTCACTATAGGG**CTACTTGTACAGCTCGTCCATGCCG	
