# Supplementary material for: Identification and characterization of catalase genes involved in the response to heat stress in Tetranychus urticae (Acari: Tetranychidae)
Source: BMC Genomics. 2025 Nov 18;26:1053. doi: 10.1186/s12864-025-12215-3 (PMC12625012; doi:10.1186/s12864-025-12215-3)
Supplement: Supplementary file 2 — Supplementary Material 2. [file 12864_2025_12215_MOESM2_ESM.pdf]

Table S2. The Ct values of RT-qPCR in *TuCATs* under short-term heat stress

Gene name	Stress temperature and duration	Ct1	Ct2	Ct3	Ct4	Ct5
TuCAT1	25 °C	22.213	22.110	22.350	22.286	22.421
	36 °C-2 h	25.056	24.717	25.004	24.864	24.997
	36 °C-4 h	24.772	24.579	24.887	24.441	24.585
	36 °C-6 h	24.323	24.333	23.922	24.085	24.167
	39 °C-2 h	24.867	24.939	24.683	24.742	24.783
	39 °C-4 h	24.469	24.215	23.986	24.057	24.192
	39 °C-6 h	23.351	23.166	23.105	23.437	23.234
	42 °C-2 h	23.012	22.811	23.263	22.671	22.946
	42 °C-4 h	22.201	22.380	22.274	22.318	22.481
	42 °C-6 h	23.366	23.209	23.429	23.245	23.279
	45 °C-2 h	24.003	24.128	23.862	23.918	24.094
	45 °C-4 h	22.882	22.911	23.051	23.194	22.948
	45 °C-6 h	23.639	23.460	23.377	23.608	23.539
TuCAT2	25 °C	19.076	19.187	19.419	19.234	19.141
	36 °C-2 h	24.859	24.589	24.678	24.618	24.822
	36 °C-4 h	24.106	24.051	24.233	24.164	23.986
	36 °C-6 h	23.238	23.533	23.250	23.341	23.187
	39 °C-2 h	23.773	23.806	23.654	23.715	23.683
	39 °C-4 h	23.374	23.503	23.285	23.417	23.483
	39 °C-6 h	22.544	22.438	22.482	22.517	22.612
	42 °C-2 h	21.927	22.116	22.016	21.951	22.038
	42 °C-4 h	22.013	21.924	21.893	21.798	21.984
	42 °C-6 h	23.751	23.469	23.585	23.541	23.366
	45 °C-2 h	24.180	24.067	23.950	23.894	24.165
	45 °C-4 h	22.193	22.210	22.420	22.394	22.439
	45 °C-6 h	22.728	22.784	22.964	22.984	22.674
α-tubulin	25 °C	18.390	18.221	17.849	18.085	17.922
	36 °C-2 h	22.042	22.375	22.178	22.263	21.951
	36 °C-4 h	21.764	22.009	21.959	22.132	22.153
	36 °C-6 h	21.453	21.511	21.722	21.625	21.493
	39 °C-2 h	21.941	21.831	21.951	21.844	21.974
	39 °C-4 h	21.503	21.801	24.464	21.489	21.159
	39 °C-6 h	20.978	20.845	20.747	20.942	20.895
	42 °C-2 h	20.067	19.517	20.177	19.863	19.933
	42 °C-4 h	20.679	19.745	20.005	19.443	19.622
	42 °C-6 h	20.708	21.149	21.680	20.943	21.338
	45 °C-2 h	22.044	22.536	22.180	22.612	22.294
	45 °C-4 h	20.619	21.228	20.097	20.349	20.492
	45 °C-6 h	21.377	20.913	20.111	20.491	20.649
