# Supplementary figures and images for: Identification and characterization of catalase genes involved in the response to heat stress in Tetranychus urticae (Acari: Tetranychidae)
Source: BMC Genomics. 2025 Nov 18;26:1053. doi: 10.1186/s12864-025-12215-3 (PMC12625012; doi:10.1186/s12864-025-12215-3)

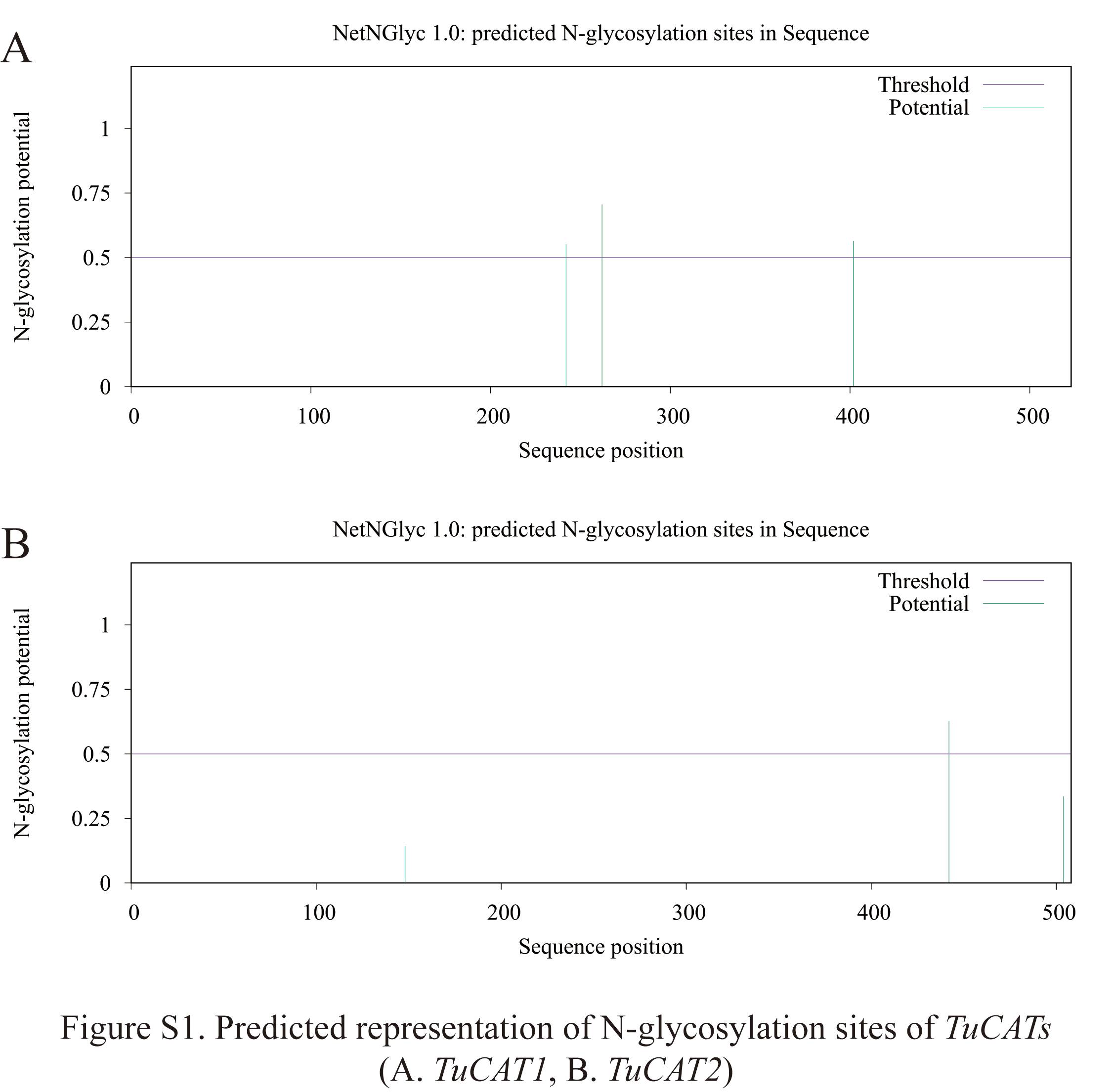

Supplement: Supplementary file 3 — Supplementary Material 3. [file 12864_2025_12215_MOESM3_ESM.jpg]

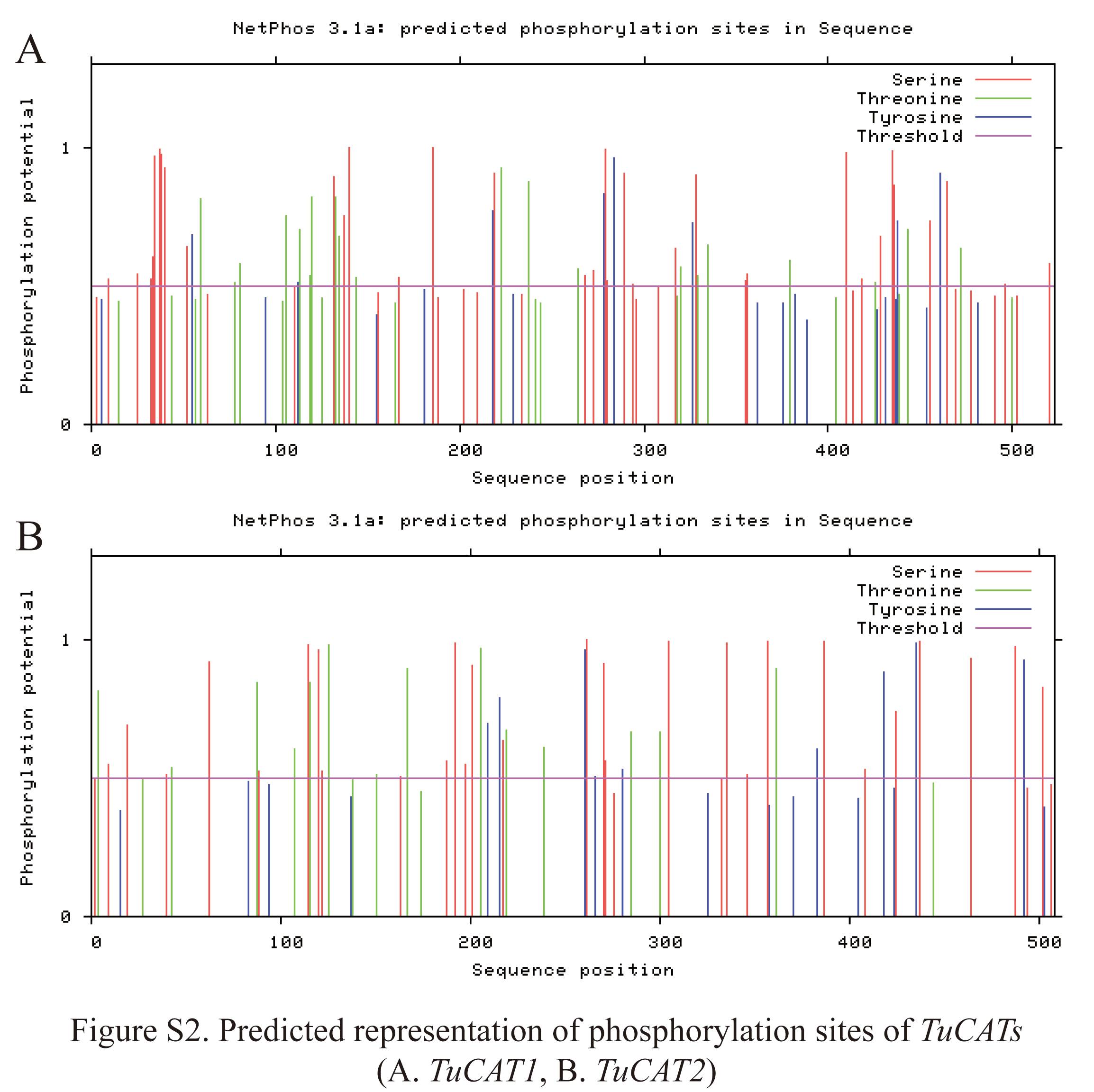

Supplement: Supplementary file 4 — Supplementary Material 4. [file 12864_2025_12215_MOESM4_ESM.jpg]

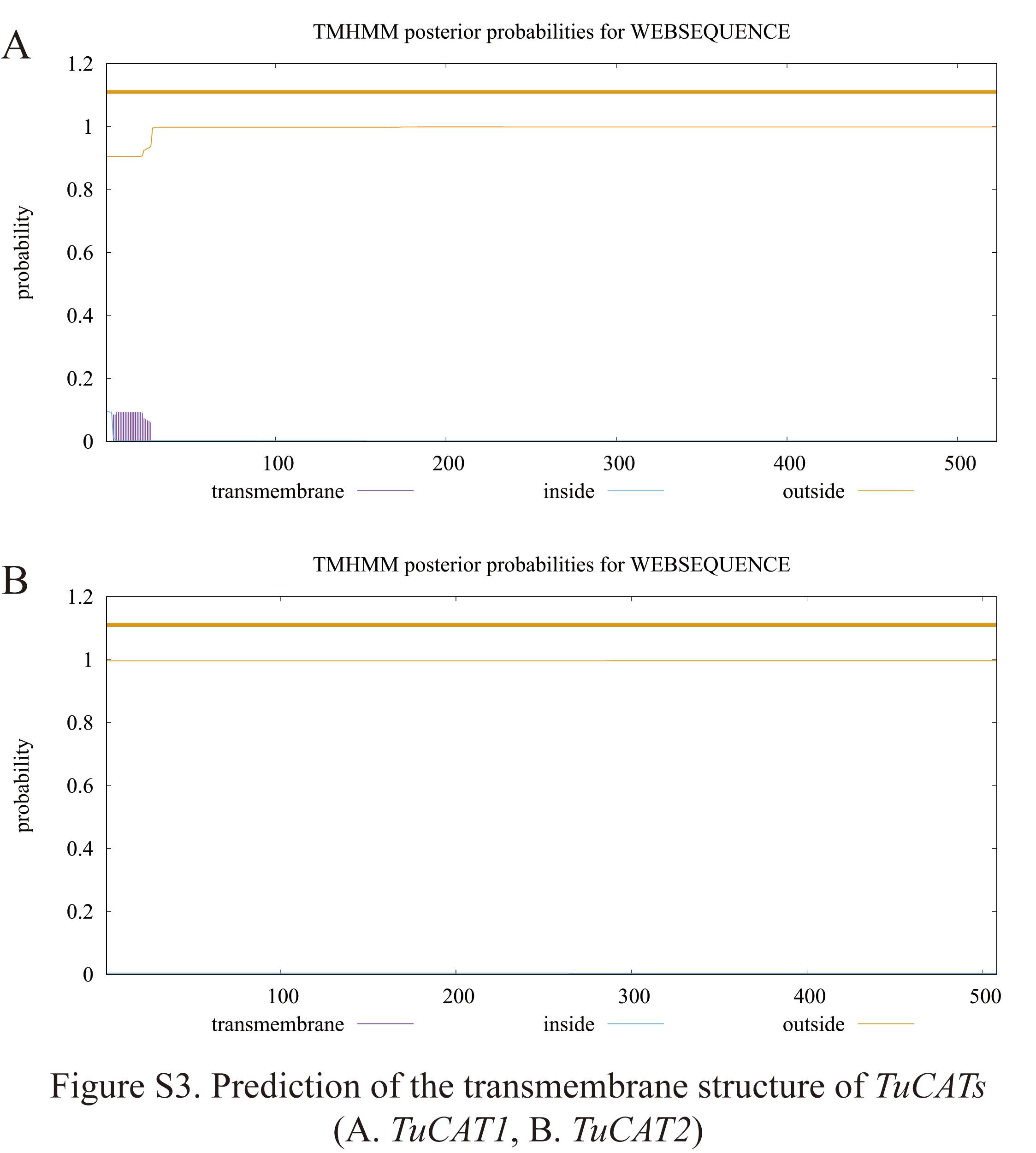

Supplement: Supplementary file 5 — Supplementary Material 5. [file 12864_2025_12215_MOESM5_ESM.jpg]

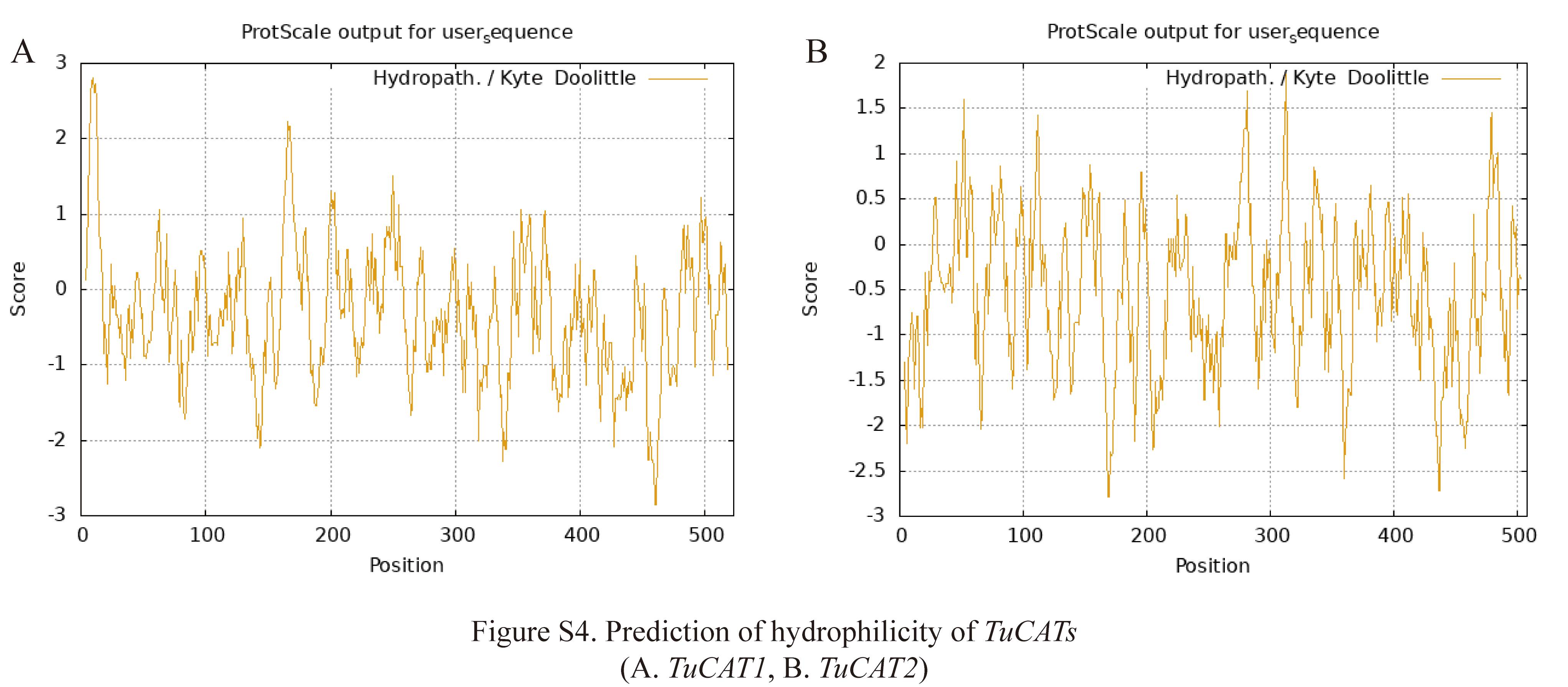

Supplement: Supplementary file 6 — Supplementary Material 6. [file 12864_2025_12215_MOESM6_ESM.jpg]
